# Perceptions of the impact of military life on relationships and Intimate Partner Violence and Abuse among UK military personnel

**DOI:** 10.1371/journal.pone.0324653

**Published:** 2025-05-28

**Authors:** Rebecca Lane, Filipa Alves-Costa, Rachael Gribble, Anna Taylor, Louise M. Howard, Nicola T. Fear, Deirdre MacManus

**Affiliations:** 1 Department of Forensic and Neurodevelopmental Sciences, Institute of Psychiatry, Psychology and Neuroscience, King’s College London, London, United Kingdom; 2 Barnet, Enfield & Haringey Mental Health NHS Trust (North London Forensic Service),; 3 King’s Centre for Military Health Research, King’s College London, Weston Education Centre, London, United Kingdom; 4 Research Department of Clinical, Educational and Health Psychology, University College London, London, United Kingdom; 5 Section of Women’s Mental Health, Institute of Psychiatry, Psychology and Neuroscience, King’s College London, London; Universiti Pertahanan Nasional Malaysia, MALAYSIA

## Abstract

Research suggests that the prevalence of Intimate Partner Violence and Abuse (IPVA) use (i.e., perpetration) and experience (i.e., victimisation) is higher among military compared to civilian populations and that military-related factors, such as deployment and deployment-related trauma, are associated with IPVA. However, the mechanisms underlying the associations between military factors and IPVA use and experience are not well understood. This study explores narratives of how military personnel perceive military life to influence IPVA within relationships. Semi-structured interviews were conducted with 40 UK military serving and ex-serving personnel (29 male, 11 female) and analysed using Framework analysis. Three superordinate themes were derived describing how elements of military life were perceived by personnel and veterans to impact on relationships and contribute to IPVA*: Demands of military work*; *Military cultural spill-over*; and *Deployment-related difficulties with psychosocial functioning and mental health.* The findings highlight risky periods for relationship conflict and IPVA, especially during reintegrations following deployments, but also show the impact of other military factors which provide significant context for IPVA. Our findings emphasise how difficulties with psychosocial functioning and communication, as well as deployment-related traumas and reintegration challenges, can influence relationships and IPVA behaviours among military personnel and veterans. Such experiences are aggravated or perpetuated by occupational demands, military socialization or institutionalisation, and the hypermasculine military environment. Targeted interventions to improve emotion regulation, conflict resolution and mentalizing skills may be especially useful for minimising harm resulting from relationship conflict and preventing IPVA.

## Introduction

Intimate Partner Violence and Abuse (IPVA), defined as “*any behaviour within an intimate relationship that causes physical, psychological or sexual harm to those in the relationship*” [1], impacts not only victim-survivors and family members [2,3], but also has substantial social and economic costs [4]. Evidence suggests the occurrence of IPVA is higher among serving and ex-serving military personnel compared with civilian populations [5–7]. In recognition of the potentially different or military-specific factors impacting the risk of IPVA within relationships with military personnel, the UK Ministry of Defence (MOD) has developed their own strategy for tackling IPVA [8,9]. This strategy notes significant gaps in the UK evidence base and while international quantitative studies have examined IPVA prevalence and associated risk factors among military populations, they fail to explore the underlying relationship dynamics and contexts in which IPVA may occur.

Research in military populations has highlighted demographic and early life factors associated with experiencing and using IPVA, including age, gender, ethnicity, level of educational achievement, employment, and adverse childhood experiences [6,10–12]. Additionally, aspects of military culture and service may contribute to relationship dissatisfaction and relationship problems, as well as IPVA experiences. Indeed, relationship dissatisfaction has been shown to be a strong predictor of IPVA in military populations [6,13]. Military training may legitimize aggression and violence within a military context [14–16], resulting in an accepted culture of aggression [17] which may bleed into other social spheres and contribute to aggressive relationships dynamics and IPVA [15]. A culture of hypermasculinity and hierarchy with unyielding rule structures has also been suggested to contribute to occupational violence spill-over [18,19], whereby gender stereotypes may be more pronounced, and dynamics of dominance and subordination may be recreated at home. Male military personnel who are victim-survivors of IPVA have highlighted how stoicism and the normalisation of violence impede both recognition of their experiences as abuse and likelihood of disclosure [20]. The higher prevalence rates of IPVA use and experience found among those serving in the Army, where such influences are arguably most intense, compared to the Royal Air Force (RAF) or Navy suggests concentration of these influences in certain military communities [5–7]. IPVA experience (i.e., victimisation) is also more prevalent among serving compared to ex-serving personnel [7], suggesting such influences wane on leaving the military.

The unique occupational demands of military service can also place stress on relationships and contribute to ‘risky’ periods for IPVA. Frequent separations and reunion may lead to increased stress among military couples [21], with personnel cycling in and out of daily family life, causing uncertainty about family roles and responsibilities [22]. Post-deployment periods have been identified as times of higher prevalence of IPVA [12,23–25], due to communication challenges and relational turbulence [26,27]. Relocations may increase non-military partners’ vulnerability to IPVA through greater social isolation and dependency on personnel [23,28,29]. Despite lower rates of IPVA among veteran populations, transition out of service may increase vulnerability to IPVA, due to difficulties adjusting to civilian life and obtaining stable housing and employment [30]. Finally, as in civilian research [31–33], personnel mental health, in particular depression, anxiety, post-traumatic stress disorder (PTSD) and alcohol use, have been shown to be risk factors for both IPVA use and experience in military populations [6,10,11,34,35]. Trauma relating to deployment experiences has been shown to be associated with increased risk of IPVA use as well as IPVA experience among military personnel [5,6,25].

While prior research highlights the potential role of various factors relating to socio-demographics, military culture, trauma exposure and deployment-related mental health, much of the international research to date has used quantitative methods to examine use of IPVA by personnel, with a focus on combat-related mental health difficulties. It has largely overlooked other important aspects of military service such as training and culture [6], as well as IPVA experiences of personnel, especially among men [7]. Recent quantitative research in UK military personnel found that risk factors for IPVA use and experience overlap [6]. However, the mechanisms underlying the associations between military factors and relationship conflict and both IPVA use and experience are not well understood and there is a limit to the depth of understanding of these mechanisms that can be offered by quantitative research. MacManus et al [6] also found bidirectional IPVA is common among serving and ex-serving UK military personnel, which, in addition to the risk of potential misclassification of perpetrators and victim-survivors in studies which do not consider the pattern and direction of relationship violence [34], highlights the need for research to examine both IPVA use and experience jointly, rather than considering each in isolation. In-depth qualitative explorations of personnel narratives of how broader military life (not just military deployment) influences relationships and abusive behaviour are needed in order to address these gaps and provide avenues for future research.

### Research aims

This study aimed to explore the perceived influence of aspects of military life on relationships and IPVA use and experience among UK military personnel and veterans (henceforth personnel). The study was guided by the following research questions:

1) How do personnel who have reported experience/use of IPVA perceive their military service (e.g., military culture, occupational demands, and operational deployments) to have affected their intimate relationships?
2) How do personnel who have reported experience/use of IPVA perceive their military service, e.g., military culture, nature of the work, and operational deployments, to have affected IPVA behaviours occurring within these relationships?

## Methods

### Study design

This study formed part of a wider mixed-methods programme aimed at better understanding IPVA use and experience among UK serving and ex-serving military personnel and civilian partners [6,23,25,36–39]. This study presents military personnel perceptions of how their military service affected their intimate relationships and influenced the occurrence of IPVA within these relationships.

### Definitions

In this paper, IPVA use refers to perpetration of IPVA towards an intimate partner, whilst IPVA experience refers to IPVA victimisation perpetrated by an intimate partner. Bidirectional IPVA describes relationships with mutual violence, whereby both partners use IPVA against the other, and therefore both also experience IPVA.

### Recruitment and data collection

Participants were recruited between 9th February and 4th September 2018 from a subsample of phase 3 respondents of the King’s Centre for Military Health (KCMHR) Health and Well-being Cohort Study [40]. Those who endorsed either perpetration of IPVA or IPVA victimisation, or both, in the 12 months previous in the phase 3 survey [6] and who consented to follow up were invited to take part via email and postal letter.

The topic guide explored participant perceptions of the impacts of military life on intimate relationship(s) and IPVA occurring within these relationships. Example questions include: *Were there specific aspects of military life or life post military, which made your relationship easier/more difficult?* Individual interviews were held over the telephone, lasting between one and two hours. All interviews were audio-recorded and transcribed verbatim using pseudonyms. Participants were given £25 compensation.

### Participants

Forty participants, 29 men and 11 women, took part in the study. Participant ages ranged from 24 to 65 years and the majority described themselves as White (Table 1). Two participants described IPVA within same-sex relationships. Most participants were ex-serving Army personnel with histories of deployment who had served as Non-Commissioned Officers.

**Table 1:** Military personnel demographics, relationship information and military characteristics.

	*n*
**Age** (years)	
*<35*	7
*35-49*	22
*50+*	11
**Ethnicity**	
*Minority ethnic group*	4
*White*	36
**IPVA reported** ^*^	
*Reporting IPVA victimisation*	28
*Reporting IPVA perpetration*	16
*Reporting bidirectional IPVA*	30
**Dual military relationship reported**	
*Yes*	16
*No*	24
**Branch**	
*Royal Navy/Royal Marines*	7
*Royal Air Force (RAF)*	11
*Army*	22
**Serving status**	
*Ex-serving (veteran)*	31
*Serving*	9
**Engagement status**	
*Regular*	33
*Reservist*	7
**Rank** (at time of interview or leaving service)	
*Officer*	7
*Non-Commissioned Officer (NCO)*	30
*Other rank*	3
**Length of service** (years)	
*5–14*	19
*15–24*	11
*25+*	10
**Deployment experience** ^**^	
*Deployed*	35
*Not deployed*	5

* Some participants reported different IPVA patterns across different relationships. As such, these are not mutually exclusive.

** Deployment experience does not include detail on whether military personnel held combat roles on deployment, although participant narratives would suggest this was common.

All participants had endorsed IPVA use and experience in the KCMHR cohort study within the last 12 months [6], including more severe IPVA. Participants described IPVA experiences across different relationships, sharing a range of patterns, severity and types of IPVA (see [37]).

### Data analysis

Data analysis was conducted using Framework analysis [41], which employs an organised structure of inductively (bottom-up) and deductively (top-down) derived themes. During the first stage of *transcription* and *familiarisation* with the data, authors read through each interview transcript to get an overall sense of the interview content. The second stage involved identifying important points and labelling recurring ideas in the data through open coding. These codes or labels were then organised to construct an initial *thematic framework* by grouping similar codes into broader themes (indexing). *A priori* themes from the literature and interview schedule were introduced into the coding framework, such as ‘relocation’, ‘separation’, ‘alcohol’ and particular types of IPVA behaviours, however were retained only where supported by participant narratives. During the *charting* stage, the framework was continually refined by moving between data and themes and summarised into a table with participant data organised under the relevant themes. Finally, during *mapping,* themes and patterns were identified and confirmed by again checking data and holding discussions among the research team. Comparisons across sub-groups (e.g., gender, IPVA use or experience, branch of service, serving status, rank) were considered and reported where relevant.

### Public and Patient Involvement (PPI)

The study benefitted from PPI consultation with military researchers, IPVA researchers and services, mental health researchers and services, members of the Armed Forces and civilians with personal experience of IPVA by their military (ex)partners at different stages of the research process. This helped inform the interview protocol and develop the framework. A PPI event generated feedback and insight on the findings as a form of participant narrative verification and validation.

### Ethical approval

Ethical Committee approval was obtained from MOD Research Ethics Committee (823/MODREC/17). Prior to involvement, participants received study information and provided informed written consent. All participants were offered the opportunity to discuss any concerns following their interview with an independent clinician and were signposted to support services.

### Reflexivity statement

All authors are White European, female, and have undertaken postgraduate studies. Authors have no current or previous affiliations to the MOD. It is important to reflect that author characteristics and pre-conceptions of the military and/or of IPVA may have influenced participant responses, affected the way the interviews were conducted and how the analysis was approached. However, the non-military status of interviewers was considered likely to reduce barriers to disclosing issues with the military institution. Analysis was conducted by three co-authors under the supervision of two others and organised into a Framework to promote transparency, with initial themes and sub-themes repeatedly interrogated. Principles of reflective practice and consultation with senior researchers, practitioners with expertise in military families research and/or IPVA, and civilian victim-survivors were used throughout the study. This allowed the team to explore author perspectives and discuss data generation and management, enhancing trustworthiness and recognition of bias through investigator triangulation [42].

## Findings

Most participants identified aspects of military life and culture they believed negatively affected their relationships and contributed to IPVA; these ranged from perceptions of increased stress on relationships, contributing to unhealthy dynamics and conflict between couples, to attributions of IPVA. Three themes were derived from the data which describe how elements of military life were perceived to impact on relationships and IPVA; *Demands of military work*; *Military cultural spill-over*; and *Deployment-related difficulties with psychosocial functioning and mental health* (Table 2).

**Table 2:** Themes and subthemes.

Themes	Subthemes	Codebook	Exemplar	RQ
1. *Demands of military work*	i. *Work -relationship conflict*	Participant alludes to conflicting priorities and demands of work and home life affecting their relationships positively or negatively.	*I end up taking [work stress] out on her, thinking that, for some reason… she is to blame, when she is obviously not… [P1]*	1
	ii. *Separations*	Participant refers to military-related separations and its relevance to relationship difficulties.	*I think most of the arguments that we had were because we just did not see each other at all. […] when we did see each other, it just felt like our time was really pressured and strained. [P11]*	1, 2
	iii. *Reintegrations into family life*	Participant refers to re-adjustment post separation and the perceived effect on their relationships and communication.	*[My partner] would often say that she had got into a new routine with the [children] whilst I was away, and it was like having someone come in and invade that routine. [P29]*	1
2. *Military cultural spill-over*	i. *Socialisation and the social context*	Participant makes direct/indirect reference to the relevance of the military social context and their relationships and communication.	*I think we have both left the military as warrant officers, so we both have that aggressive response to situations. We don’t tend to take slight on any of our responses. We tend to both default on fight. [P34]*	2
	ii. *Institutionalisation*	Participant describes institutionalised learning stemming from their military occupation and its effect on their interactions with others.	*Just pure anger [when she didn’t do what I asked or wanted] because I didn’t understand why she wasn’t being a soldier like the soldiers had been soldiers, because, when I told them to do something, they were doing it. Of course, it was difficult to switch off sometimes, a bit like Jekyll and Hyde. [P29]*	2
	iii. *The hypermasculine environment*	Participant makes direct/indirect reference to the hypermasculine environment.	*I was told, if I stayed in the military, I would be butch; if I cut my hair too short, I would look like a man; if I drank pints of beer rather than half pints that wasn’t ladylike, and I would end up being overweight. He didn’t like the way I dressed. He didn’t like the fact that I was confident. I was confident back then. [P39]*	2
3. *Deployment-related difficulties with psychosocial functioning and mental health*	i. *Heightened anger and aggression*	Participant describes increased anger and aggression towards their partner following deployment.	*I think that was six months after I had come back from Afghanistan as well, so […] even if somebody just touched me in an aggressive way, I would just obviously reciprocate in the same way. […] It is almost like you can feel yourself inside getting more and more angry […] I would pick an argument then. Hell or high water, I would get an argument. [P21]*	2
	ii. *Mental health difficulties*	Participant describes directly/indirectly how mental health difficulties on deployment affected their relationships and communication.	*[After deployment], that is when I started getting nightmares and started withdrawing, just not talking about feelings and all that sort of thing. I guess that just allowed, with me being not very verbal and outspoken about what I was feeling, […] her to be controlling in that respect and get what she wanted because I was never bothered enough about it. [P13]*	2

### Theme 1: Demands of military work

Theme 1 speaks to how the specific demands of military work were perceived to place pressure on relationships, increasing relationship stress and unhealthy dynamics, and contributing to IPVA.

#### Work-relationship conflict.

Most participants spoke of work-relationship conflict, highlighting stressors which they perceived as leaving them with limited time and energy for family relationships and reducing their ability to communicate with their partner. Examples included unpredictable or long working hours, work stress, and the sensitive and demanding nature of the work. A minority of participants described efforts by the military to accommodate their relationships and family life and the benefits of financial stability and housing being provided in service.

*I end up taking [work stress] out on her, thinking that, for some reason… she is to blame, when she is obviously not… [You aren’t] able to plan your family life and your relationship around your job. We are not guaranteed any leave that we apply for… It put distrust in [partner’s] head because she perhaps thought I was holding back from […] planning things together.* [P1; Male Navy; bidirectional IPVA]

*[Being in the military] helped with [my partner] coming over here, getting visas, staying over here. They gave us a house to stay in.* [P21; Male Army; IPVA victimisation with retaliation]

Participants across all service branches explained how tensions between the demands of their military career and family life could lead to increased relationship difficulties and conflict, especially among those of higher rank. For some, especially male participants, the perceived pressure from the military to prioritise operational effectiveness and career progression was viewed to contribute to the de-prioritisation of their non-military partner and increase unhealthy relationship dynamics.

*Arguments [became] more frequent, they were more volatile. I became more dismissive of her point of view. For me it was about just doing my job and getting recognition for that and working up the ranks and feeling self-important. I had a blindfold on for a lot of my service life, not seeing the damage [to the relationship] I was doing by pursuing a career without thought for anything else.* [P29; Male Army; IPVA perpetration with victim retaliation]

The tension between the military and the family was heightened among dual-serving couples. Many explained how one partner had had to leave the military to accommodate the other’s career or manage child-care. Leaving service was reported to result in isolation and poor self-esteem among some non-serving partners, resulting in relationship conflict and resentment. This was especially prominent for female partners who had been regulars in the Army.

*We had a very difficult relationship when my wife left the Army and when I did my first two tours in Iraq, […] it caused a lot of problems in our relationship because she felt very lonely, she felt that I was leaving her.* [P10; Male Army; Dual-military relationship; bidirectional IPVA]

#### Separation.

The nature of military training and deployments was reported to result in frequent and extended periods of separation for couples, sometimes at short notice. This was especially true for dual-serving couples with conflicting deployment patterns. Ongoing separation was perceived to put significant pressure on relationships by contributing to impaired communication and emotional detachment and limiting opportunities for conflict resolution.

*I think most of the arguments that we had were because we just did not see each other at all. […] when we did see each other, it just felt like our time was really pressured and strained.* [P11; Female Navy; Dual-military relationship; IPVA perpetration with victim retaliation]

Many participants attributed their poor relationship satisfaction and relationship breakdowns solely to time spent apart due to military duties. Some reflected on how their relationship dynamics had improved since their transition out of service, with reduced conflict.

*When I look back at things, that is the main reason my marriage broke down was the amount of time we went away for.* [P7; Male Army; IPVA perpetration with victim retaliation]

*Our relationship has improved over the seven years that I have been home. […] Whereas before the argument would be the argument but then it would have carried on, it could be for quite a few days, whereas now […] if we do argue, and it is not the severity that we used to before […] [we are] straightaway afterwards, basically, apologising to each other or accepting what the other one said.* [P10; Male Army; Dual-military relationship; bidirectional IPVA]

As well as separations affecting relationship quality and dynamics, some participants believed separations contributed to IPVA within their relationships. Dissatisfaction with the participant’s military career by their non-serving partners and pressure to leave the military as a result was a common theme among those reporting experiences of victimisation, especially men.

*Both of my previous wives asked me to leave the military; they were fed up with me being in the military.* [P40; Male RAF; IPVA victimisation]

Separations were perceived to contribute to emotional insecurity (such as fears of infidelity) and increased burden for non-deploying or serving partners, leading to controlling behaviours and emotional manipulation of personnel. Separations were also reported to facilitate the experience of financial abuse for some, as partners oversaw finances while participants were away.

*I always had to be in on time. I always had to account for where I had been if I was late. […] I wasn’t allowed to go out with friends. I wasn’t really allowed to have friends. She hated my army friends, if I ever had anything to do with them.* [P23; Male Army; IPVA victimisation with retaliation]

*I would be away for sometimes a month at a time, and then come home and I would find that the bank would be empty* [P40; Male RAF; IPVA victimisation]

Despite separations escalating difficulties for some, both male and female participants reporting experience of IPVA described how separations offered an opportunity for transient escape or respite from an abusive partner.

*The military and my job allows me to focus on what I am doing and provides me with an escape [from] dealing with an otherwise stressful marriage. […] She hates [my job]. She really does hate it. […] [After deployment], it is nice to be home, but I worry about what mood [partner] will be in and how it will be between us.* [P36; Male RAF; IPVA victimisation with retaliation]

#### Reintegrations into family life.

Following separation and deployment, participants described a process of ‘acclimatising’ to their relationship and family environment. Most highlighted this time as a period of increased relationship conflict. Common post-separation relationship destabilisers were the need for space or time alone, discrepancies between each partner’s experience during the time apart, not feeling understood (including in dual-military relationships with differing roles) and being underwhelmed by their welcome home. These experiences were reported to result in participants feeling disconnected from their spouses and family unit and was associated by some with vulnerability to experiencing IPVA or attempts to reassert dominance.

*I was there to fight the wars and she was there to help support the civilians and the military afterwards. That, in itself, was an issue of friction. […] She doesn’t know what it is like to be stabbed, to be blown up, to be shot. Don’t get me wrong, I don’t want her to ever know that. But, until you experience it, how can you describe to somebody what it is like to be blown up and finding body parts all over you. […] I get quite irate with that.* [P26; Male Army; Dual-military relationship; bidirectional IPVA]

*[My partner] would often say that she had got into a new routine with the [children] whilst I was away, and it was like having someone come in and invade that routine. […] for example, they would eat when they wanted to eat, whereas, if I was at home, we would always have dinner at, say, 7 o’clock. […] I would come in… just expect that we are still in that old routine.* [P29; Male Army; IPVA perpetration with victim retaliation]

A minority described similar difficulties and increased conflict when transitioning out of service.

*When I left the army, I then came home, and that is where a lot of our problems really started. Obviously, when I was at home, I was at home all the time so we were arguing more. My wife found it very difficult to adjust with me being at home all the time.* [P10; Male Army; Dual-military relationship; bidirectional IPVA]

### Theme 2: Military cultural spill-over

Theme 2 describes participant perceptions of the role socialisation into military culture played in their relationships and how it contributed to IPVA, observing aspects of military culture and work to spill-over into their family homes.

#### Aggressive behaviours.

Many participants described difficulties managing and expressing their emotions leading to anger and impulsivity and associated these with perpetration of verbal and physical aggression within their relationships. Some participants described observing their anger building and boiling over, and that in the absence of a release for their emotions, for instance through exercise or engaging in violence in another context, observed increased conflict at home.

*I think the anger was due to the military side of things. [Working as a prison officer] was a release from that, because [the anger] would build up and build up. It would be like you would go for weeks where nothing would happen in the prison, and you could feel yourself like a boiling pot. That is when the fights would be worse when we got home.* [P24; Male Army; IPVA perpetration with victim retaliation]

Although some shared that they had aggressive traits prior to joining the military, many attributed difficulties managing anger to their military training and roles. Participants perceived the military environment to legitimise and encourage their use of aggressive communication styles, learned aggressive responses and a heightened sensitivity to threat, which contributed to more aggressive interactions with their partners.

*I [haven’t been] physical at anyone when I have been angry, but I will do things like punching doors and garages and brick walls... You are being trained to kill and everything, and the way in which [the military] do that is just a very aggressive life to live… joining the army quite early, you are at a very impressionable age in your life. I just soaked it all up like a sponge... I have learnt to be angry, I have learnt how to fight* [P13; Male Army; IPVA victimisation with retaliation]

Participants associated increased/learned aggression with poor conflict resolution strategies, where conflict was either met with avoidance (walking away) or mirrored with increased aggression. This was a prominent issue among Army personnel, Non-Commissioned Officers, dual-military relationships, and those who had deployed. Adopting a defensive and aggressive response to conflict was also particularly prominent among those in relationships in which both partners engaged in aggressive behaviours.

*We disagree on a few things here and there, and sometimes I do feel quite raged. The only way that I do things is to just walk away, but she takes that as something quite rude.* [P21; Male Army; IPVA victimisation with retaliation]

*I think we have both left the military as warrant officers, so we both have that aggressive response to situations. We don’t tend to take slight on any of our responses. We tend to both default on fight. So, any confrontations, we are quite confrontational back.* [P34, Female Army; Dual-military relationship; bidirectional IPVA]

Aggressive behaviours were also linked by some to their, or their partner’s, emersion into the military drinking culture, leading to increased conflict and antagonistic behaviour due to lowered inhibitions and amplification of aggressive tendencies.

*He was never physically violent towards me, but, on two or three occasions where he did, drunk, and absolutely very, very angry, he caused a lot of damage to the house, throwing things and breaking glasses and pulling pictures off walls and throwing chairs. […] I think the way that the military use alcohol, whether it encouraged [his] behaviours, but it certainly made it very easy, that kind of alcohol use to be treated like normal, and actually encouraged.* [P16; Female Army; Dual-military relationship; bidirectional IPVA]

*I would be more readily picking an argument [after consuming alcohol], and I would pick it over anything I could find, just to have an argument.* [P24; Male Army; IPVA perpetration with victim retaliation]

Minimisation of IPVA behaviours was common among participants, potentially due to a tolerance, minimisation or normalisation of violence and abusive behaviours. This was especially apparent in descriptions of behaviours consistent with non-physical forms of IPVA, which were often not perceived to be abusive by the participant but were also apparent in reports of physical IPVA.

*He wasn’t abusive or anything. He just used to come back and he used to just go, ‘I really hate you. I wish I’d never married you,’ and, ‘you’re just a frigid bitch.’ It would just be verbal.* [P20; Female RAF; Dual-military relationship; bidirectional IPVA]

*She has hit me before. I have had a glass HP bottle over my kneecap when we first got married. That would be [all].* [P5; Male RAF; bidirectional IPVA]

Some female military personnel who reported perpetrating physical IPVA also showed a tendency to minimise physically violent behaviour towards their partners. In addition, there was a sense that masculine ideals exacerbated some male participants’ reluctance to perpetrate or retaliate physical aggression towards their female partner.

*She would throw a drink all over me and shout at me in public. She would throw food at me… I have had situations where she has pushed me into the road… I can’t do anything because it is a woman… although I wanted to do exactly the same, I couldn’t…* [P8; Male RAF; IPVA victimisation]

#### Institutionalisation.

Across all branches of the military, participants’ need for order and obedience developed through their military career, as well as high expectations of themselves and others, could contribute to feelings of impatience and anger towards partners. Such perceptions were linked to increased use of abusive language, reduced tolerance of others (especially civilians), and attempts to obtain control and enforce rank dynamics within the home. This was described as worse during periods of increased stress, such as post-deployment and in periods of mental ill-health, see Theme 1, subtheme 3 and Theme 3.

*Just pure anger [when my partner didn’t do what I wanted] because I didn’t understand why she wasn’t being a soldier like the soldiers had been soldiers, because, when I told them to do something, they were doing it. Of course, it was difficult to switch off sometimes, a bit like Jekyll and Hyde.* [P29; Male Army; IPVA perpetration with victim retaliation]

Authoritarian and rigid attitudes and the military hierarchy were said to spill over into relationship dynamics at home on a spectrum from unhealthy interactions to controlling and abusive behaviours. This was significant among dual-military couples and was a particular difficulty for participants with civilian partners, who are not accustomed to military culture.

*The feeling that I outranked him, he still used to feel on the back foot, and I think, at times, when we were arguing, you say stupid things to put them down, when you lash out.* [P11; Female Navy; Dual-military relationship; IPVA perpetration with victim retaliation]

*Throughout every relationship I have had, especially any civilian relationship, they always seem to think I come across as quite abrupt and sharp sometimes. They feel like I am instructing them. […] without realising it, I come across as very authoritative* [P32; Female Army personnel; bidirectional IPVA]

Military hierarchy and status were also believed to affect behaviours or dynamics within relationships due to a heightened sensitivity to a perceived loss of status, particularly after leaving service. Socialisation in a culture of obedience and following orders could also contribute to vulnerability to victimisation by coercive control for some by their civilian partner due to a lack of agency arising from their time in service.

*I came out of the army where I was the second in command of the regiment at one point, and [now my partner is of higher ‘rank’ than me]. […] I will become quite difficult with [my partner] in terms of picking on things and criticising her role within the senior leadership team […] I think I am just jealous of the fact that I am now a private soldier again in many respects.* [P29; Male Army; IPVA perpetration with victim retaliation]

*She told me that everything I have ever achieved […] is nothing, and she just thinks that I wasted my time in the army, and the army just used me. So basically just putting down. […] I think that, having been in the army for so long, […] I tell my wife, if she wants something done, let me know. […] I tend to just follow orders still, and I wait for orders still.* [P21; Male Army; IPVA victimisation with retaliation]

#### The hypermasculine environment.

Narratives of both men and women revealed how working in a military “macho” culture was perceived to have widespread influence on their relationships, impacting their emotional functioning and ability to connect with their partners.

*During operations the last thing you want to be doing is showing emotions […] So you are tougher […] You are cold and hard at those times […] The [partners] didn’t like it, when they are trying to be all lovey-dovey. […] It could [cause more arguments].* [P27; Male Army; IPVA victimisation]

For female participants, working and socialising in a male-dominated environment was perceived to contribute to relationship difficulties and was associated with experiences of victimisation by partners, especially emotional abuse and coercive control.

*I was told, if I stayed in the military, I would be butch; if I cut my hair too short, I would look like a man; if I drank pints of beer rather than half pints that wasn’t ladylike, and I would end up being overweight. He didn’t like the way I dressed. He didn’t like the fact that I was confident. I was confident back then.* [P39; Female Army; Dual-military relationship; IPVA victimisation]

*He finds it quite hard the fact that I am in the military and it is a very male-dominated environment. […] He doesn’t like me socialising with a lot of people on ship. So, I just don’t anymore.* [P11; Female Navy; Dual-military relationship; IPVA victimisation with retaliation]

### Theme 3: Deployment-related difficulties with psychosocial functioning and mental health

Theme 3 summarises how participants perceived mental health and psychosocial difficulties experienced following deployments to affect their relationships and IPVA.

#### Heightened anger and aggression.

Participants reported experiencing increased anger and aggression when returning to the family environment following separations, especially combat deployments. This was described to affect their interactions with, and their ability to relate to, their partners and contributed to an increase in frequency and severity of arguments. Feelings of restlessness, anger and default aggression and increased alcohol use to cope were described and linked to participant difficulties with adjustment. This was a more prominent theme among male participants and Army personnel.

*We were arguing about something and had had a few drinks. I think I came home late, and she wanted to know where I was. Obviously, she started arguing, and I was just in bed lying down. She came and tried to obviously shake me, and I punched her. […] After Afghanistan] if somebody just touched me in an aggressive way, I would just obviously reciprocate in the same way. […] you can feel yourself inside getting more and more angry […] I would pick an argument then. Hell or high water, I would get an argument.* [P21; Male Army; IPVA victimisation with retaliation]

#### Mental health difficulties.

Post deployment psychological difficulties were frequently reported to influence behaviours within relationships, especially among men who deployed in combat roles. Some participants reported symptoms suggestive of PTSD, such as hypervigilance and hyperarousal, night terrors or flashbacks, which contributed to relationship conflict in different ways due to changes in emotional and interpersonal functioning. Some perceived their mental health difficulties to result in increased irritability and more aggression in their relationships.

*The day after [experiencing flashbacks], all day I would feel guilty for being here […] If I was with [partner], that is when I would be really grumpy and pick a fight, an argument. […] I wanted to feel better myself, and to have an argument almost was like a way of detracting from it. […] The anger was building up and up and up, and, at the time, I didn’t know how to deal with it.* [P24; Male Army; IPVA perpetration with victim retaliation]

Others reported depressive symptoms and social withdrawal, which manifested in passivity and avoidance of conflict. Participants described the latter to frustrate non-military partners and trigger or escalate arguments, which could result in experiences of victimisation.

*When I came back from Afghanistan, […] I was talking in my sleep a lot; I would wake up screaming, things like that… When you do come back from a warzone… You don’t take things as seriously as some people would. […] In that respect, that probably caused some friction. […] I took a non-engagement stance; I didn’t engage in the argument, […] so then it escalates.* [P9; Male Army; IPVA victimisation]

*[After deployment], that is when I started getting nightmares and started withdrawing, just not talking about feelings and all that sort of thing. I guess that just allowed, with me being not very verbal and outspoken about what I was feeling, […] her to be controlling in that respect and get what she wanted because I was never bothered enough.* [P13; Male Army; IPVA victimisation with retaliation]

## Discussion

This study explored serving and ex-serving UK military personnel’s perceptions of how military service, life and culture affect intimate relationships and IPVA. Three themes were generated: *Demands of military work*; *Military cultural spill-over*; and *Deployment-related difficulties with psychosocial functioning and mental health*. Building on findings from previous research which has explored how aspects of military life may affect relationships [12,21,23,24,27,30], conflicting priorities of military vs family, work stress and separations were perceived to promote conflict and unhealthy relationship dynamics within relationships and lead to reduced relationship satisfaction and communication between couples. These were particularly prominent issues among dual-serving couples, an under-researched group [43]. These findings are consistent with civilian research which has found aggression is more likely to arise between couples who are under stress [44]. Civilian research has also shown work-to-home interference increases anger towards others at home among those with low job control [45], particularly relevant to a hierarchical institution like the military, and supports the application of general strain theory [46] in explaining the perpetration of violence at an individual level.

Resentment and emotional insecurity from civilian partners due to dependency on their military partners, a perceived lack of agency, and a perceived prioritisation of the military over the family was also described, echoing other research on civilian partners of military personnel [28,47]. In cases where IPVA victimisation was described from civilian partners, separations and deployments could be an escape from IPVA in their relationship. Military welfare should be alert to the potential of patterns of behaviour as indicators of IPVA in their communities.

Other factors were reportedly more directly linked with negative or IPVA behaviours within relationships, such as the use of aggressive communication styles. Supporting previous research which described occupational violence spill-over within military populations [15–17,19], participants reported aspects of military training and culture to affect their interpersonal functioning at home. Many reported difficulties with anger, adopting aggressive styles of communication and an intensified response to confrontation, some of which was attributed to military training. These factors could contribute to *de novo* conflicts or escalations in relationship conflict and appeared especially prominent in participants who served or are serving in the Army, providing important context for understanding quantitative associations between IPVA and serving in the Army [5–7]. Furthermore, many participant narratives reveal how elements of military hierarchical structure, such as having high expectations and use of power and subordination to maintain control, can lead to behaviours within relationships which could be perceived as controlling; these behaviours may be minimised or overlooked as part of the military way of being. Indeed, these issues have been identified in the narratives of civilian victim-survivors of IPVA occurring within intimate relationships with UK military personnel [23].

Similar difficulties with conflict resolution and emotion regulation have been described in samples of police officers [48], whereby negative emotions/attitudes and authoritarian attitudes were found to affect perceptions of threats and contribute to heightened aggressive responses and IPVA. This supports the ‘angry aggression’ theory of violence which states that a subculture of angry aggression arises in environments of high arousal and under conditions of social isolation, reinforced through the concentration of feedback loops [49]. Significantly, the minimisation or normalisation of violence observed in participant narratives may contribute to a worsening in the severity of violence and prolongation of IPVA, bidirectional IPVA or female perpetrated IPVA, also highlighted by Taylor et al. [20]. The difficulties with interpersonal communication described by participants in the current study are consistent with research among UK male military personnel, which found that the hypermasculine environment may impact on personnel’s ability to communicate their emotions, difficulties, or distress [50]. This was found to have implications for seeking help for mental health difficulties and likely impacts military personnel’s ability and inclination to seek help for IPVA. Given the findings of the present study and the known high prevalence of both IPVA use and experience among military serving and ex-serving personnel [6], more must be understood of military personnel’s views and experiences of help-seeking for IPVA.

Our findings add to existing research into the mechanisms by which deployment-related trauma or mental health difficulties are associated with relationship difficulties and IPVA [5,6,10,11,25,27,51]. Many participant narratives highlight greater potential for military cultural spill-over during times of transition and reintegration, and suggest a need for increased preparation, decompression time and support, especially following deployment in combat zones. Of note, participant narratives showed that the same factors could be linked to IPVA use or experience, or both, in different relationships. This was especially apparent in the discussion of mental health difficulties and interpersonal communication difficulties. In this study, symptoms linked to externalizing behaviours, such as hyperarousal and emotion dysregulation, appeared more prominent in the narratives of those reporting perpetration of IPVA; whilst symptoms linked to internalizing behaviours, such as withdrawal, appeared to be associated with a heightened vulnerability to IPVA victimisation. These findings may help contextualise and explain quantitative findings of associations between mental health difficulties or disorders, such as PTSD, depression or anxiety, and both IPVA victimisation and perpetration [6,25,34,35]. Although noted, alcohol use was not as prominent a theme as expected given its significant association with both IVPA experience and use in the literature [6,10,11,34]. Further in-depth exploration is needed to better understand these underlying mechanisms and context for how mental health difficulties and psychosocial functioning more broadly are associated with IPVA.

### Strengths and limitations

This study provides much needed understanding into the complex ways in which military service and culture are perceived by military personnel to impact and shape their intimate relationships and contribute to IPVA occurring within these relationships. This is facilitated by qualitative exploration, which provides deep, contextual insights into lived experiences, uncovering nuances, complexities, and perspectives that quantitative data alone cannot capture. The study importantly gives voice to male victim-survivors, a group currently under-represented [7] and provides information on the difficulties experienced by dual-military couples. These findings also complement research exploring the perceptions and experiences of civilian victim-survivors, whose partners were service or ex-serving military personnel [23], by providing the perspectives of military personnel.

Limitations should be noted. Although Framework Analysis lends itself to subgroup comparison [52], notable differences between groups were not frequently observed. This may be reflective of the complexity of disentangling the various factors that can influence experiences of IPVA beyond socio-demographic or military characteristics, as well as the high levels of bidirectional IPVA observed in the sample. Additionally, participants largely reported less severe experiences of IPVA within their intimate relationships. While this aligns with findings that less severe forms of IPVA predominate [6], social desirability bias may have influenced participants’ willingness to disclose more distressing experiences during interviews, particularly those associated with feelings of shame. This may be compounded with possible minimisation and normalisation of IPVA within the occupational and hypermasculine military context [38].

Attempts to mitigate the risk of social desirability bias were made in the recruitment process and study design. Firstly, participants were recruited from a subsample of respondents of Phase 3 of the KCMHR Health and Well-being Cohort Study [40], all of whom had endorsed perpetration of IPVA or IPVA victimisation, or both, within the past year. The interview schedule allowed participants to elaborate on their experiences, providing details and context of IPVA behaviours in each significant relationship reported, with prompts used to clarify IPVA types, severity and frequency. Additionally, interviews were conducted by non-military researchers with previous experience of conducting sensitive interviews using a non-judgmental approach, which likely reduced barriers to disclosing IPVA behaviours and appraisals of the military institution. While reluctance to share sensitive experiences remain potential limitations, qualitative methods were intentionally selected to understand meaning, context, and lived experiences of military personnel which are rarely platformed and explored.

It is also important to highlight that despite efforts made to recruit diversely across gender, ethnicity, sexuality, branch, and IPVA history, the present research recruited a predominantly White British heterosexual male sample, all of whom had previously deployed. Whilst this sample is in some respects demographically representative of personnel in military service (military personnel surveys indicate that most personnel across all services were of White ethnicity (93.1%) and were male (90.3%) [53]) and is generous in sample size, the restricted range of narratives on which our findings are based must be acknowledged. Further qualitative investigation is warranted to explore differences in experiences across groups, including dual military couples, LGBTQ+ couples, female personnel, and personnel from other ethnic backgrounds, to provide a more comprehensive understanding of how a military career and context is perceived to influence intimate relationships and IPVA behaviours occurring within these relationships.

### Implications and conclusion

With research evidence supporting claims that IPVA is higher in the UK military compared to the civilian population [6], our findings provide valuable insight into the aspects of military service which are perceived be most influential on these outcomes. These should be utilised to inform future domestic abuse policy developments or revision of the current MOD Domestic Abuse Strategy [8,9] and policy [54]. With a broader focus on relationships rather than just IPVA, our findings facilitate valuable understanding of how military life and culture can negatively impact intimate relationships. This research describes the contexts in which IPVA may arise, complementing quantitative research examining IPVA among military personnel [6] and qualitative civilian partner research [23]. Such understanding is essential to the UK Armed Forces’ effort to provide better support to military families [6,8,9].

The findings highlight risky periods for relationship conflict and IPVA, especially during reintegrations following deployments, adding to previous research [51] and supporting findings from recent quantitative work showing significant associations between deployment-related trauma and mental health problems and IPVA perpetration [25]. Our findings complement this by highlighting the influence of other aspects of military service and culture which provide significant context for IPVA. Culture change is needed in the military community to engender attitudes which are more conducive to and supportive of healthy relationships among personnel, e.g., more progressive attitudes to gender, masculinity, and the balance between military priorities and relationship/family needs. Support is needed for personnel to adapt their emotional and behavioural responses from military to civilian and family settings in order to tackle the problem of interpersonal aggression within the home. Significantly, many participants describe aspects of military life and culture which they felt impacted on their emotional and interpersonal functioning, in turn affecting their ability to connect and appropriately communicate with partners. It is of note that spill-over of aggression into the home sphere and problems with anger management are not highlighted in the MOD Domestic Abuse Strategy [8,9] and is not a specific focus in any more recent policy developments. Interventions aimed at improving emotion regulation, conflict resolution and mentalizing skills to minimise harm resulting from relationship conflict and preventing IPVA may be especially useful.

Greater awareness is needed of periods of increased risk of IPVA by military personnel, such as reintegrations post separation, the peri-deployment period and the transition to civilian life, with targeted efforts made to improve identification and support, and reduce barriers to help-seeking for those at risk or who have experienced IPVA during these periods. Education on IPVA should be available to personnel and military families as part of training/well-being packages, for instance on HIVEs in military bases, especially in anticipation of key risk periods such as the peri-deployment period and transition out of service. Importantly, the findings stress the importance of difficulties with emotional, psychological and behavioural functioning associated with mental health disorders or difficulties, emphasising the need for a multidisciplinary, interagency, collaborative approach to tackling IPVA within military communities. Further research is needed to investigate the experiences of male military personnel victim-survivors, LGBT+ couples, and victim-survivors from minority ethnic groups.

Our findings provide valuable insight into the aspects of military service which are perceived to impact negatively on relationships and influence the occurrence of IPVA and should be utilised to support current efforts by the Ministry of Defence to improve their support provision to military families and prevent, reduce and improve management of IPVA.
